# Patients with unmet social needs are at higher risks of developing severe long COVID-19 symptoms and neuropsychiatric sequela

**DOI:** 10.1038/s41598-024-58430-y

**Published:** 2024-04-02

**Authors:** Anna Eligulashvili, Megan Darrell, Moshe Gordon, William Jerome, Kevin P. Fiori, Seth Congdon, Tim Q. Duong

**Affiliations:** 1 Department of Radiology, Albert Einstein College of Medicine and Montefiore Medical Center, 1300 Morris Park Avenue, Bronx, NY 10461 USA; 2 Department of Pediatrics, Albert Einstein College of Medicine and Montefiore Medical Center, Bronx, NY USA; 3 Department of Medicine, Albert Einstein College of Medicine and Montefiore Medical Center, Bronx, NY USA

**Keywords:** PASC, Long covid, Covid symptoms, Fatigue, Shortness of breath, Viral infection, Risk factors

## Abstract

This study investigated long COVID of patients in the Montefiore Health System COVID-19 (CORE) Clinics in the Bronx with an emphasis on identifying health related social needs (HRSNs). We analyzed a cohort of 643 CORE patients (6/26/2020–2/24/2023) and 52,089 non-CORE COVID-19 patients. Outcomes included symptoms, physical, emotional, and cognitive function test scores obtained at least three months post-infection. Socioeconomic variables included median incomes, insurance status, and HRSNs. The CORE cohort was older age (53.38 ± 14.50 vs. 45.91 ± 23.79 years old, *p* < 0.001), more female (72.47% vs. 56.86%, *p* < 0.001), had higher prevalence of hypertension (45.88% vs. 23.28%, *p* < 0.001), diabetes (22.86% vs. 13.83%, *p* < 0.001), COPD (7.15% vs. 2.28%, *p* < 0.001), asthma (25.51% vs. 12.66%, *p* < 0.001), lower incomes (53.81% vs. 43.67%, 1^st^ quintile, *p* < 0.001), and more unmet social needs (29.81% vs. 18.49%, *p* < 0.001) compared to non-CORE COVID-19 survivors. CORE patients reported a wide range of severe long-COVID symptoms. CORE patients with unmet HRSNs experienced more severe symptoms, worse ESAS-r scores (tiredness, wellbeing, shortness of breath, and pain), PHQ-9 scores (12.5 (6, 17.75) vs. 7 (2, 12), *p* < 0.001), and GAD-7 scores (8.5 (3, 15) vs. 4 (0, 9), *p* < 0.001) compared to CORE patients without. Patients with unmet HRSNs experienced worse long-COVID outcomes compared to those without.

## Introduction

Post-acute sequelae of SARS-CoV-2 infection (PASC), also referred to as long COVID, has emerged as a significant public health concern in part because of the sheer number of individuals infected by SARS-CoV-2^1–4^. PASC includes, but are not limited to, anxiety, depression, post-traumatic stress disorder, and other neurological, psychiatric, and cognitive symptoms^5–8^.

COVID-19 has disproportionately affected marginalized communities, amplifying the inequalities they face in accessing healthcare, resources, and socioeconomic stability^9,10^. Factors such as limited access to quality healthcare, higher rates of underlying health conditions, overcrowded living conditions, and employment in essential but higher-risk jobs have all contributed to the heightened impact of COVID-19 on vulnerable populations^11,12^. A few studies have shown that patients with lower socioeconomic status or unmet health related social needs (HRSNs) are more likely to be infected by SARS-CoV-2 and have worse acute clinical outcomes^13–18^. These vulnerable populations are also at higher risk of developing long COVID but are less studied^19^. Common HRSNs include food and housing, utilities, out of pocket healthcare costs, child/adult care, and healthcare transportation. Examining the relationship between HRSNs and PASC could shed light on the likely challenges faced by this population and could help to address the nuanced needs of individuals experiencing prolonged health consequences post-COVID infection^20^.

The Bronx in New York City was hit hard by the first wave of COVID-19 and by multiple subsequent surges of infection from different variants^21^. Montefiore Health System is the largest health care provider in Bronx County, serving a diverse patient population, including many patients with low socioeconomic status. In June 2020, Montefiore Medical Center established two COVID-19 Recovery and Engagement (CORE) Clinics for COVID-19 survivors with protracted symptoms. A detailed evaluation protocol was implemented to assess physical, emotional, and function across the neurological and psychiatric domains for patients suspected to have long COVID.

In this paper, we reported long-COVID symptoms, physical, emotional, and cognitive function of CORE patients from June 26th, 2020 and February 24th, 2023. Outcomes were interpreted with emphasis on their relationship with socioeconomic data which included median incomes, insurance status, and HSRNs. The objective of this study was to identify the prevalence of HSRNs as a risk factor for long COVID symptoms incidence and severity in a diverse and underserved population of CORE patients.

## Methods

### Data sources

This study was approved by the Einstein IRB (IRB# 2021–13658) with a waiver of informed consent. The Montefiore Health System included a large number of hospitals and outpatient clinics in the Bronx and surrounding towns. This is a retrospective observational cohort study of COVID-19 adult patients with protracted symptoms who were referred to our COVID-19 Recovery and Engagement (CORE) clinics. Eligible patients were at least 18 years of age who had probable or confirmed COVID-19 infection and were experiencing new or continued symptoms greater than 4 weeks after initial infection. Patients who came to the CORE clinics included both patients who were hospitalized and not hospitalized for COVID-19. CORE patients could come from anywhere and could be referred to by any health provider. The CORE clinics were also advertised in the local communities. There were 643 patients who came to the CORE clinics for COVID-19 related medical issues between June 26th, 2020 and February 24th, 2023.

As an aside, brief comparisons of patient profiles were also made for all COVID-positive (COVID +) cohort which consisted of 52,089 Montefiore patients who returned alive three months after initial COVID + diagnosis date over the same period. The date of the first positive reverse transcription polymerase chain reaction (RT-PCR) was extracted as the date of COVID-19 diagnosis. For CORE patients without positive RT-PCR results in their chart, the date of COVID-19 infection is recorded by CORE physicians in the doctor’s note and collected via chart review.

### Data abstraction

De-identified health data were obtained for research after standardization to the Observational Medical Outcomes Partnership (OMOP) Common Data Model (CDM) version 6. OMOP CDM represents healthcare data from diverse sources, which are stored in standard vocabulary concepts^19^, allowing for the systematic analysis of disparate observational databases, including data from the electronic medical record (EMR), administrative claims, and disease classifications systems (e.g., ICD-10, SNOWMED, LOINC, etc.). ATLAS, a web-based tool developed by the Observational Health Data Sciences and Informatics (OHDSI) community that enables navigation of patient-level, observational data in the CDM format, was used to search vocabulary concepts and facilitate cohort building. Data were subsequently exported and queried as SQLite database files using the DB Browser for SQLite (version 3.12.0). To ensure data accuracy, our team performed extensive cross validation of all major variables extracted by manual chart reviews on subsets of patients^13–18^.

### Demographics and comorbidities

Age, sex, BMI, combined race ethnicity, median household income quintile, and insurance status were extracted from patient EMR. Age and BMI at the time of or closest to COVID-19 diagnosis were recorded. Income data was obtained by matching patient’s zip code to median household income as reported by the census, and then assigning quintiles based on the cohorts’ combined incomes. Preexisting comorbidities included hypertension, chronic obstructive pulmonary disease (COPD), asthma, diabetes mellitus, congestive heart failure (CHF), and chronic kidney disease (CKD) that were designated by ICD10 codes at admission or prior.

This retrospective study was approved by the Einstein-Montefiore Institutional Review Board (#2021–13658). This study has an exemption for informed consent and a HIPAA waiver and was performed in accordance with relevant guidelines and regulations.

### COVID-19 disease severity

COVID-19 disease severity was categorized into non-hospitalized, hospitalized (general floor), and hospitalized (critically ill). All hospitalized patients admitted to the intensive care unit (ICU) or placed on invasive mechanical ventilation (IMV) were considered critically ill.

### Vaccination status

The vaccination status of CORE patients at the time of COVID-19 infection was recorded by CORE physicians in the doctor’s note and collected via chart review. Vaccination status could not be collected for COVID + patients given the inconsistent coding in Epic and large sample size requiring chart review.

### Health related social needs

The majority of CORE patients completed the Montefiore-Einstein Social Determinants of Health Screen, which is a standardized instrument developed by Health Leads Toolkit (https://healthleadsusa.org/) and adapted by Montefiore-Einstein’s Office of Community and Population Health in 2018. This screening tool is provided during routine clinical visits across the health system and is integrated into the EHR. The responses were not required (it was voluntary). The screener asks about the following social need categories: housing, food insecurity, utilities, health transportation, medications, child or elderly care, legal services, family stress and safety^22^. The number of responses to date is about 250,000.

### Long COVID physical symptom questionnaires

All CORE patients were surveyed for the presence of various post-infection symptoms, labeled as “Symptoms Present at 1st Core Visit.” CORE patients additionally completed a revised Edmonton Symptom Assessment System (ESAS-r) where they scored various symptoms from 0 (no symptoms or best overall wellbeing) to 10 (the worst level of symptom imaginable or worst overall wellbeing). The median, first quartile (Q1), and third quartile (Q3) of each ESAS-r symptom severity was calculated.

### Long COVID behavioral symptom questionnaire

CORE patients were assessed for depression and anxiety using the Patient Health Questionnaire (PHQ-9) Questionnaire and Generalized Anxiety Disorder (GAD-7) Questionnaire, respectively. Questionnaire results were reported as median (Q1, Q3), in addition to incidence of various respective provisional diagnosis categories.

### Statistical analysis

Analysis was performed by comparing CORE patients with all other COVID-19 patients. Analysis was also performed between patients with and without unmet social needs. Analysis of group differences employed χ^2^ tests for categorical variables and unpaired t-tests for continuous variables via the statistical libraries in SPSS and RStudio, respectively. Mann–Whitney U tests were performed for median comparisons. When comparing differences of categorical variables across three groups, χ^2^ tests were used to identify group differences and ad hoc pairwise tests were used to identify the specific pairwise group difference. Statistics were not adjusted for multiple comparison and no variables were controlled for due to the exploratory nature of the study. *P* < 0.05 was considered statistically significant.

## Results

**Figure 1:**
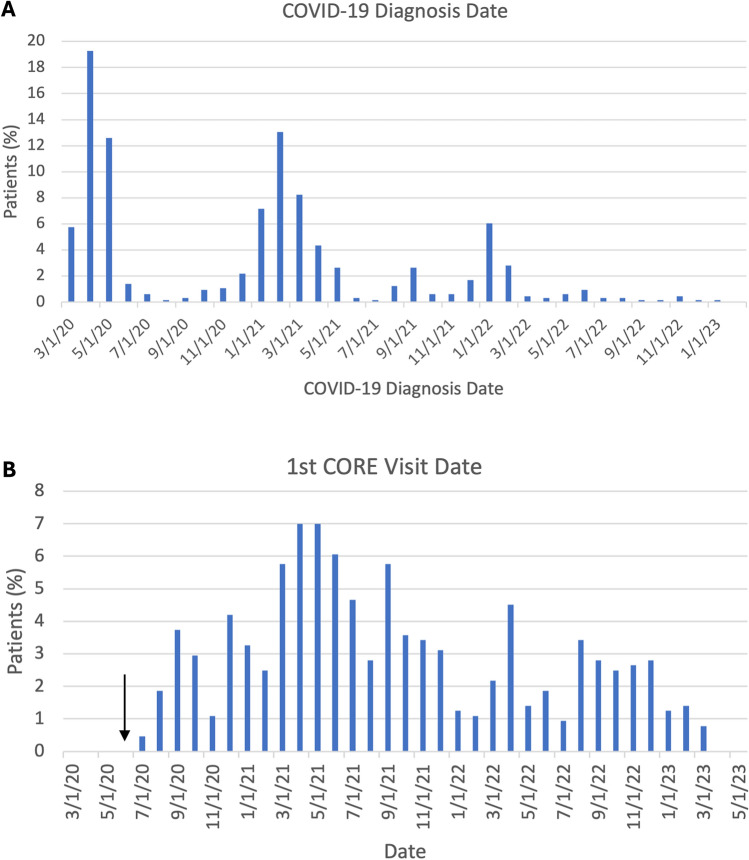
(**A**) Distribution of COVID-19 diagnosis date of CORE patients. (**B**) Distribution of 1st CORE visit dates for CORE patients. CORE opened on 06/26/2020, indicated by the black arrow. The number of patients were expressed as percentage (i.e., all % of patients across all months add up to 100%).

Figure 1 displays a histogram of date of COVID-19 infection and 1st CORE visit. Relatively more CORE patients were infected earlier in the pandemic compared to later in the pandemic. More CORE patients visited early in the pandemic compared to later in the pandemic.

Table 1 shows the demographic, disease severity, and socioeconomic profiles of CORE patients. The average age was 53.38 ± 14.50, majority female (72.47%), Hispanic (46.50%), unvaccinated (83.67%), and with a median household income quintile less than $55,275.80 (53.81%).

**Table 1:** CORE patient profiles at diagnosis.

	CORE (N = 643)
Age, years old	53.83 ± 14.50
Female	466 (72.47%)
BMI	31.80 ± 7.64
Combined race and ethnicity	
White, not Hispanic	62 (9.64%)
Black, not Hispanic	170 (26.44%)
Hispanic	299 (46.50%)
Other	112 (17.42%)
Comorbidities	
Hypertension	295 (45.88%)
COPD	46 (7.15%)
Asthma	164 (25.51%)
Diabetes Mellitus	147 (22.86%)
CHF	32 (4.98%)
CKD	36 (5.60%)
COVID-19 disease severity	
Hospitalized (General Floor)	147 (22.86%)
Hospitalized (Critically Ill)	50 (7.78%)
Vaccination status	
Unvaccinated	538 (83.67%)
Partially vaccinated	5 (0.78%)
Fully Vaccinated	65 (10.11%)
Fully Vaccinated with Booster	27 (4.20%)
Median household income quintile	
1 ($55,275.80)	346 (53.81%)
2 ($69,938.40)	151 (23.48%)
3 ($88,767.60)	82 (12.75%)
4 ($115,314.60)	22 (3.42%)
5 ($250,000)	27 (4.20%)
Insurance status	
Private	212 (32.97%)
Medicaid	204 (31.73%)
Medicare	96 (14.93%)
Uninsured	45 (7.00%)
Care management organization > Private	77 (11.98%)
Other	7 (1.09%)
Unmet social needs	N = 369
0	259 (70.19%)
1 +	110 (29.81%)

Values reported as mean ± standard deviation (SD) or N (%). The median time frame from acute COVID to CORE clinic visit was 194 days.

As an aside, we compared CORE patient profiles only with all COVID + patient profiles (Supplemental Table 1). Compared to the *all* COVID + cohort, CORE patients were older (53.38 ± 14.50 versus (vs.) 45.91 ± 23.79 years old, *p* < 0.001), had higher BMI (31.80 ± 7.64 vs. 28.34 ± 7.70, *p* < 0.001), and more likely to be female (72.74% vs. 56.86%, *p* < 0.001). Except for White, not Hispanic (9.64% vs. 12.42%, *p* = 0.033) and Hispanic (46.50% vs. 39.84%, *p* = 0.001), there were no statistically significant differences in combined race and ethnicity (*p* > 0.05) between the two groups. With respect to comorbidities, CORE patients had higher proportions of hypertension (45.88% vs. 23.28%, *p* < 0.001), COPD (7.15% vs. 2.28%, *p* < 0.001), asthma (25.51% vs. 12.66%, *p* < 0.001), and diabetes mellitus (22.86% vs. 13.83%, *p* < 0.001), but no statistically significant difference in CHF or CKD (*p* > 0.05). CORE patients had slightly decreased hospitalization (general floor) rates (22.86% vs. 23.86%, *p* < 0.001), but no difference in critical illness (*p* > 0.05). CORE patients were also primarily unvaccinated at the time of COVID-19 infection (83.67%).

With respect to socioeconomic data, CORE patients had higher proportions of 1st (lowest) quintile median household income as compared to the COVID + cohort (53.81% vs. 43.67%, *p* < 0.001). Additionally, CORE patients were more likely to have private insurance (32.97% vs. 26.19%, *p* < 0.001) or care management organization (11.98% vs. 3.97%, *p* < 0.001), but also less likely to use Medicaid (31.73% vs. 41.01%, *p* < 0.001) and Medicare (14.93% vs. 19.51%, *p* = 0.004). Overall, there were significantly more CORE patients with unmet social needs (29.62% vs. 18.49%, *p* < 0.001).

Supplemental Fig. 1 presents the HRSN questionnaire results for CORE versus COVID + groups broken down by individual unmet need. CORE patients had statistically significant greater incidence of concerns about housing quality (11.96% vs. 5.57%, *p* < 0.001), housing situation (11.14% vs. 5.02%, *p* < 0.001), money for food (8.97% vs. 5.86%, *p* = 0.046), utilities shut threat (5.98% vs. 3.21%, *p* = 0.012), and legal help (5.43% vs. 2.77%, *p* = 0.008) as compared to the COVID + group.

The results of the long COVID physical symptom and behavioral questionnaires for CORE patients are shown in Table 2. The most common symptoms were dyspnea (47.43%), fatigue (39.04%), decreased exercise tolerance (27.84%), and brain fog/cognitive issues (27.53%). According to the ESAS-r Questionnaire, the most severe symptoms were tiredness (median 6; IQR (3, 8)), worsened overall wellbeing (5 (1.25, 7)), and shortness of breath (4 (0, 7)). On average, PHQ-9 and GAD-7 questionnaires showed CORE patients experienced mild depression (8 (3, 14)) and no or little anxiety (4 (1, 10)).

**Table 2:** Long COVID symptom questionnaire results stratified by COVID-19 disease severity.

	CORE (N = 643)	Non-hospitalized (N = 446)	Hospitalized general floor (N = 147)	Hospitalized critically ill (N = 50)
Symptoms present at 1st CORE visit				
Dyspnea	305 (47.43%)	185 (41.48%) (41.48%)**	80 (54.42%)^^	40 (80.00%)^+++^
Fatigue	251 (39.04%)	163 (36.55%) (36.55%)	59 (40.14%)^	29 (58.00%)^++^
Decreased Exercise Tolerance	179 (27.84%)	100 (22.42%) (22.42%)**	54 (36.73%)	25 (50.00%)^+++^
Brain Fog/Cognitive Issues	177 (27.53%)	120 (26.91%) (26.91%)	39 (26.53%)	18 (36.00%)
Abnormal Smell/Taste	85 (13.22%)	66 (14.80%)	16 (10.88%)	3 (6.00%)
Cough	75 (11.66%)	47 (10.54%)	15 (10.20%)^^	13 (26.00%)^++^
Chest Pain	66 (10.26%)	53 (11.88%)	9 (6.12%)	4 (8.00%)
Headache	57 (8.86%)	49 (10.99%)*	7 (4.76%)	1 (2.00%)^+^
Joint Pain	50 (7.78%)	30 (6.73%)	15 (10.20%)	5 (10.00%)
Palpitations	47 (7.31%)	30 (6.73%)	12 (8.16%)	5 (10.00%)
Back Pain	42 (6.53%)	33 (7.40%)	8 (5.44%)	1 (2.00%)
Lightheadedness/Dizziness	39 (6.07%)	27 (6.05%)	8 (5.44%)	4 (8.00%)
Post-exertional Malaise	15 (2.33%)	11 (2.47%)	4 (2.72%)	0 (0.00%)
ESAS-r Responses (scale of 0–10, best to worst)	N = 390–397	N = 249–256	N = 100–101	N = 40–41
Tiredness ‡	6 (3, 8)	6 (3, 8)	5 (1.75, 7.25)	3 (0, 7)^++^
Overall Wellbeing ‡	5 (1.25, 7)	5 (2, 7)	5 (1, 7)	2 (0, 5)^+^
Shortness of Breath ‡	4 (0, 7)	4 (0, 7)	4 (0, 7)	3 (1, 6)
Pain ‡	4 (0, 6)	4 (0, 6)	4 (0, 7)^	1 (0, 4)^++^
Anxiety ‡	3 (0, 7)	4 (0, 7)*	2 (0, 6)	0 (0, 3)^++^
Drowsiness ‡	3 (0, 6)	3 (0, 6)	2 (0, 5)	1.5 (0, 5)
Depression ‡	2 (0, 5)	2 (0, 5)	1 (0, 6)	0 (0, 3)^+^
Lack of Appetite ‡	0 (0, 4)	0 (0, 4)	0 (0, 4)^^^	0 (0, 0)^+++^
Nausea ‡	0 (0, 1)	0 (0, 1.5)	0 (0, 0)	0 (0, 0)^++^
PHQ-9 (0–27)	N = 401	N = 259	N = 101	N = 41
Overall	8 (3, 14)	9 (4, 14)	8 (3, 15)^^	3 (1, 6)^+++^
Normal or Minimal Depression (0–4)	132 (32.92%)	67 (15.02%)*	29 (32.22%)^	119 (54.29%)^+++^
Mild Depression (5–9)	102 (25.44%)	76 (17.04%)	18 (20.00%)	8 (22.86%)
Moderate Depression (10–14)	77 (19.20%)	60 (13.45%)	15 (16.67%)	2 (5.71%)^++^
Moderately Severe Depression (15–19)	56 (13.97%)	34 (7.62%)	19 (21.11%)	3 (8.57%)
Severe Depression (20 +)	34 (8.48%)	22 (4.93%)	9 (10.00%)	3 (8.57%)
GAD-7 (0–21)	N = 398	N = 259	N = 98	N = 41
Overall	4 (1, 10)	5 (2, 11)**	3 (0, 10)	1 (0, 5)^+++^
No or Little Anxiety (0–4)	200 (50.25%)	115 (25.78%)	25 (47.17%)	13 (52.00%)^++^
Mild Anxiety Disorder (5–9)	80 (20.10%)	60 (13.45%)	16 (30.19%)	4 (16.00%)
Moderate Anxiety Disorder (10–14)	60 (15.08%)	43 (9.64%)	12 (22.64%)	5 (20.00%)
Severe Anxiety Disorder (15–21)	58 (14.57%)	41 (9.19%)	0 (0.00%)	3 (12.00%)

Values reported as median (Q1, Q3) or N (%). *p < 0.05, **p < 0.01, ***p < 0.001 between non-hospitalized and hospitalized (general floor). ^*p* < 0.05, ^^*p* < 0.01, ^^^*p* < 0.001 between hospitalized (general floor) and hospitalized (critically ill). ^+^*p* < 0.05, ^++^*p* < 0.01, ^+++^*p* < 0.001 between non-hospitalized and hospitalized (critically ill).

CORE patients were broken down by disease severity into non-hospitalized (N = 446), general floor (N = 147), and critically ill (N = 50). Across disease severity groups, higher incidence of dyspnea, fatigue, decreased exercise tolerance, and cough incidence were associated with higher disease severity. Incidence of normal or minimal depression and anxiety both increased as disease severity worsened.

The demographic, disease severity, and socioeconomic profiles of CORE patients without and with unmet HRSNs are presented in Table 3**.** There were no statistically significant differences in age, female sex composition, BMI, combined race and ethnicity, or comorbidities (all *p* > 0.05). Disease severity and vaccination status was also not significantly different between groups (*p* > 0.05). Patients with unmet needs had lower incidence of 5th quintile median household income (0.91% vs. 5.79%, *p* = 0.035) and higher Medicaid use (43.64% vs. 26.64%, *p* = 0.001) compared to patients without unmet needs.

**Table 3:** Demographics by without and with unmet social needs in CORE patients.

	Without unmet needs (N = 259)	With unmet needs (N = 110)	*P*-value
Age, years old	54.06 ± 14.38	51.60 ± 12.23	0.09
Female	192 (74.13%)	77 (70.00%)	0.06
BMI	31.82 ± 7.32	33.38 ± 7.27	0.41
Combined Race and Ethnicity			
White, not Hispanic	34 (13.13%)	8 (7.27%)	0.10
Black, not Hispanic	72 (27.80%)	38 (34.55%)	0.19
Hispanic	107 (41.31%)	52 (47.27%)	0.29
Other	46 (17.76%)	12 (10.91%)	0.09
Comorbidities			
Hypertension	116 (44.79%)	52 (47.27%)	0.66
COPD	16 (6.18%)	10 (9.09%)	0.31
Asthma	55 (21.24%)	28 (25.45%)	0.37
Diabetes Mellitus	47 (18.15%)	24 (21.82%)	0.41
CHF	7 (2.70%)	5 (4.55%)	0.36
CKD	13 (5.02%)	6 (5.45%)	0.86
COVID-19 disease severity			
Hospitalized (General Floor)	59 (22.78%)	31 (28.18%)	0.26
Hospitalized (Critically Ill)	20 (7.72%)	8 (7.27%)	0.88
Vaccination status			
Unvaccinated	203 (78.38%)	86 (78.18%)	0.96
Partially Vaccinated	4 (1.54%)	0 (0.00%)	0.19
Fully Vaccinated	35 (13.51%)	17 (15.45%)	0.62
Fully Vaccinated with Booster	14 (5.41%)	5 (4.55%)	0.73
Median household income quintile			
1 ($55,275.80)	139 (53.67%)	69 (62.73%)	0.10
2 ($69,938.40)	57 (22.01%)	22 (20.00%)	0.66
3 ($88,767.60)	35 (13.51%)	11 (10.00%)	0.35
4 ($115,314.60)	8 (3.09%)	4 (3.64%)	0.78
5 ($250,000)	15 (5.79%)	1 (0.91%)	0.035
Insurance status			
Private	105 (40.54%)	34 (30.91%)	0.08
Medicaid	69 (26.64%)	48 (43.64%)	0.001
Medicare	33 (12.74%)	14 (12.73%)	0.99
Uninsured	21 (8.11%)	3 (2.73%)	0.055
Care management organization > Private	27 (10.42%)	9 (8.18%)	0.50
Other	3 (1.16%)	1 (0.91%)	0.83

Values reported as mean ± standard deviation (SD) or N (%).

Table 4 compares the long COVID physical symptoms and behavioral questionnaires stratified by patients without and with social needs. Only brain fog/cognitive issues were higher in patients with unmet needs (42.73% vs. 32.05%, *p* = 0.049) compared to patients without 0 unmet needs. In the ESAS-r responses, patients with unmet needs did experience more severe symptoms compared to patients without unmet needs, with the top five severe ones being tiredness (7 (4.25, 8) vs. 5 (0,7), *p* = 0.002), worsened overall wellbeing (5 (4, 7) vs. 4 (1, 6), *p* < 0.001), shortness of breath (5 (1, 7) vs. 3 (0, 6), *p* = 0.025), pain (5 (2, 7) vs. 3 (0, 6), *p* < 0.001), and anxiety (5 (1, 8) vs. 2 (0, 5), *p* < 0.001). Patients with unmet needs additionally experienced worsened depression (12.5 (6, 17.75) vs. 7 (2, 12), *p* < 0.001) and anxiety (8.5 (3, 15) vs. 4 (0, 0), *p* < 0.001) as compared to patients without unmet needs according to the PHQ-9 and GAD-7, respectively. CORE patients with unmet needs had increased rates of clinically severe depression (15.60% vs. 5.93%, *p* = 0.003) and severe anxiety disorder (28.44% vs. 8.39%, *p* < 0.001).

**Table 4:** Long COVID symptom questionnaire results stratified by without and with unmet social needs in CORE patients.

	Without unmet needs (N = 259)	With unmet needs (N = 110)	*P*-value
Symptoms present at 1st CORE visit			
Dyspnea	137 (52.90%)	55 (50.00%)	0.61
Fatigue	117 (45.17%)	52 (47.27%)	0.71
Decreased exercise tolerance	65 (25.10%)	24 (21.82%)	0.50
Brain fog/cognitive issues	83 (32.05%)	47 (42.73%)	**0.049**
Abnormal smell/taste	35 (13.51%)	17 (15.45%)	0.62
Cough	32 (12.36%)	10 (9.09%)	0.36
Chest pain	21 (8.11%)	12 (10.91%)	0.38
Headache	19 (7.34%)	10 (9.09%)	0.56
Joint pain	23 (8.88%)	7 (6.36%)	0.41
Palpitations	24 (9.27%)	7 (6.36%)	0.35
Back pain	19 (7.34%)	8 (7.27%)	0.98
Lightheadedness/Dizziness	17 (6.56%)	9 (8.18%)	0.57
Post-exertional malaise	7 (2.70%)	3 (2.73%)	0.98
ESAS-r responses (scale of 0–10, best to worst)			
Tiredness	5 (0, 7)	7 (4.25, 8)	**0.002**
Overall wellbeing	4 (1, 6)	5 (4, 7)	**< 0.001**
Shortness of breath	3 (0, 6)	5 (1, 7)	**0.025**
Pain	3 (0, 6)	5 (2, 7)	**< 0.001**
Anxiety	2 (0, 5)	5 (1, 8)	**< 0.001**
Drowsiness	2 (0, 5)	5 (0, 8)	**< 0.001**
Depression	0 (0, 5)	5 (0.25, 8)	**< 0.001**
Lack of appetite	0 (0, 3)	2 (0, 5)	**< 0.001**
Nausea	0 (0, 0)	0 (0, 2)	**0.007**
PHQ-9 (0–27)			
Overall	7 (2, 12)	12.5 (6, 17.75)	**< 0.001**
Normal or minimal depression (0–4)	97 (38.34%)	19 (17.43%)	**< 0.001**
Mild depression (5–9)	68 (26.88%)	22 (20.18%)	0.17
Moderate depression (10–14)	49 (19.37%)	21 (19.27%)	0.98
Moderately severe depression (15–19)	24 (9.49%)	30 (27.52%)	**< 0.001**
Severe depression (20 +)	15 (5.93%)	17 (15.60%)	**0.003**
GAD-7 (0–21)			
Overall	4 (0, 9)	8.5 (3, 15)	**< 0.001**
No or little anxiety (0–4)	177 (61.89%)	35 (31.11%)	**< 0.001**
Mild anxiety disorder (5–9)	50 (17.48%)	22 (20.18%)	0.95
Moderate anxiety disorder (10–14)	35 (12.24%)	21 (19.27%)	0.20
Severe anxiety disorder (15–21)	24 (8.39%)	31 (28.44%)	**< 0.001**

Values reported as median (Q1, Q3) or N(%). n = 259 for all variables except overall wellbeing (n = 258) in the cohort without needs. n = 110 for all variables in the cohort with unmet needs.

Significant values are in [bold].

## Discussion

This study investigated the patient characteristics and prevalence of long COVID symptoms in our CORE clinics between June 26th, 2020 and February 24th, 2023 with emphasis on the potential influence of unmet HRSNs. The major findings are: (i) most CORE patients are infected during the early waves of COVID-19 and were largely unvaccinated, (ii) COVID-19 survivors report a wide range of severe long-COVID symptoms across the physical, emotional, and cognitive health domains, (iii) some COVID-related symptoms (i.e., dyspnea, fatigue, decreased exercise tolerance, and cough) show higher incidence among more severe COVID-19 patients but these symptoms were not limited to hospitalized or critically ill patients, and (iv) patients with unmet HRSNs have similar demographics (except lower percentage of high income bracket and higher percentage with Medicaid), but experience similar rates of physical and psychological long-COVID symptoms compared to patients without unmet HRSNs, but with greater severity of symptoms.

More CORE patients were infected during the early waves. These peak infection periods matched the reported SARS-CoV-2 infection spikes in New York metropolitan areas^23^. Vaccination and treatments were limited during the earlier waves of the pandemic and thus it is likely that these patients infected in earlier waves experienced more severe disease courses^24–26^. Our data indicated that most CORE patients were in fact unvaccinated at the time of infection. Patients infected in later waves likely had less severe disease course and long-term outcomes as vaccines and COVID-19 treatments became more available.

The high incidences of dyspnea, fatigue, brain fog, decreased exercise tolerance, and shortness of breath reported in this study are consistent with the literature^27,28^. Goertz et al.^29^ found that fatigue, dyspnea, headache, and chest tightness were the four most common persistent symptoms 3-months after infection. Huang et al.^30^ reported that fatigue or muscle weakness was by far the most common symptom hospitalized COVID-19 patients at 6-months followed up. Carfi et al.^31^ found fatigue, dyspnea, and joint pains to be the three most prevalent symptoms in COVID survivors about 2 months after their COVID-19 hospitalization. These symptomologies are not surprising given many COVID-19 patients were often discharged with major medical referrals^32,33^. The incidence of dyspnea, fatigue, decreased exercise tolerance, and cough increased as disease severity worsened, in agreement with other papers^34–36^.

Additionally, CORE patients broadly showed signs of mild anxiety and mild depression. Surprisingly, the severity of anxiety and depression was inversely correlated to COVID-19 disease severity. Other studies have similarly found that after COVID-19 infection, a significant number of patients self-reported anxiety and depression^37,38^. One study found that most COVID-19 survivors had mild depression, specifically using the PHQ-9 questionnaire, in agreement with our study. As anxiety and depression are known risk factors for developing PASC^39^, it is concerning that the majority of CORE patients screened positive for clinically significant anxiety and depression.

Some, but not all, CORE patients showed more long-COVID symptoms and abnormal physical, emotional, and cognitive scores. More severe COVID-19, as measured by hospitalization in the general floor or ICU/IMV interventions, showed higher incidence of long COVID symptoms compared to the non-hospitalized cohort. It is striking that many non-hospitalized patients had substantial symptoms and abnormal scores. Although non-hospitalized patients who visited our CORE clinics were more likely to exhibit symptoms given the nature of their visit, these data suggested that long COVID is not limited to severely ill COVID-19 patients. A recent prospective study demonstrates similar findings, in which patients with high respiratory symptomatic burden 3-months post-COVID were associated with high disease severity^40^. Previous studies have reported individuals with mild symptoms from SARS-CoV-2 infection (i.e., not requiring hospitalization) or asymptomatic infection may also be susceptible to general and neurological PASC^41–43^.

CORE patients with and without unmet HRSNs had similar baseline demographics, comorbidities, and disease severity in our cohort, except patients with unmet social needs had lower proportion of 5^th^ quintile income and more were on Medicaid as expected. However, the incidence of severe depression and anxiety was nearly tripled in patients with unmet HRSNs as compared to patients without. Patients with unmet HRSNs also experienced more severe long COVID symptoms compared to those without.

It is likely that the persistent stress associated with these unmet needs is increasingly understood as a pivotal factor, amplifying the susceptibility to and severity of long-term consequences from COVID-19. It is well known that sustained, chronic stress has several deleterious effects on normal physiology. It sets off a chain reaction within the body, sparking inflammatory responses and prompting intricate alterations in gene expression, known as epigenetic modifications^44^. These modifications, in turn, have the potential to significantly impact health outcomes, potentially worsening the long-term effects of COVID-19.

Moreover, persistent structural barriers in accessing quality health care services, particularly preventative care, places vulnerable populations at a distinct disadvantage. The higher prevalence of pre-existing health conditions among marginalized groups, often due to systemic inequalities, acts as a significant contributor to the severity of long COVID outcomes. The cumulative effect of these intersecting factors serves to magnify the disproportionate toll that COVID-19 exacts on historically marginalized populations, highlighting the urgent need for targeted interventions addressing these underlying socioeconomic disparities.

This study has several limitations. Our findings were limited to COVID-19 survivors who came to our CORE clinics and who were more likely to have more severe COVID-19 symptoms, and thus our cohort was not representative of the general population. Findings also rely on the assumption that COVID-19 controls did not seek medical care outside of the Montefiore system for long-COVID symptoms, as this data would not be available. Multicenter and prospective studies are needed to validate these findings and achieve broader generalization. It was not possible to definitively distinguish abnormalities that were due to COVID-19, pre-existing or worsened by COVID-19 disease. SDOH data were only available for a subset of patients because these questionnaires were incorporated in 2018^45^ with the administration and responses being encouraged but voluntary. Long-term outcomes may have been affected by vaccination status and reinfection. Long COVID-19 is time dependent post infection^46–49^. It is possible that symptom score related to long-COVID is dependent the duration of time post infection. A previous work has identified no change in clinical variables at 3–6 months follow-up^50^.

## Conclusions

COVID-19 patients have a wide range of severe long COVID symptoms across the physical, emotional, and cognitive health domains, and these symptoms are not limited to patients who were hospitalized or critically ill. Patients with unmet social needs are more likely to experience exacerbated long COVID outcomes, furthering the widening health disparity. These findings call attention to the impacts of health disparity on socioeconomically vulnerable patients with long COVID. This pandemic has underscored the urgent need to address systemic issues and work towards equitable healthcare and support systems for all.

### Supplementary Information

Supplementary Information.

## Data Availability

The data used in this study are available upon request to the corresponding author.
