# Supplementary material for: Patients with unmet social needs are at higher risks of developing severe long COVID-19 symptoms and neuropsychiatric sequela
Source: Sci Rep. 2024 Apr 2;14:7743. doi: 10.1038/s41598-024-58430-y (PMC10987523; doi:10.1038/s41598-024-58430-y)
Supplement: Supplementary file 1 — Supplementary Information. [file 41598_2024_58430_MOESM1_ESM.docx]

**Supplemental Table 1.** *CORE and all COVID+ patient cohort profiles at diagnosis. Values reported as mean ± standard deviation (SD) or N (%). The median time frame from acute COVID to CORE clinic visit was 194 days.*

|  | **CORE**  **(N=643)** | ***All* COVID+**  **(N=52,089)** | **P-value** |
| --- | --- | --- | --- |
| Age, years old | 53.83±14.50 | 45.91±23.79 | **<0.001** |
| Female | 466 (72.47%) | 29,619 (56.86%) | **<0.001** |
| BMI | 31.80±7.64 | 28.34±7.70 | **<0.001** |
| Combined Race and Ethnicity |  |  |  |
| White, not Hispanic | 62 (9.64%) | 6,472 (12.42%) | **0.033** |
| Black, not Hispanic | 170 (26.44%) | 14,712 (28.24%) | 0.312 |
| Hispanic | 299 (46.50%) | 20,751 (39.84%) | **0.001** |
| Other | 112 (17.42%) | 10,026 (19.25%) | 0.242 |
| **Comorbidities** |  |  |  |
| Hypertension | 295 (45.88%) | 12,177 (23.38%) | **<0.001** |
| COPD | 46 (7.15%) | 1,189 (2.28%) | **<0.001** |
| Asthma | 164 (25.51%) | 6,595 (12.66%) | **<0.001** |
| Diabetes Mellitus | 147 (22.86%) | 7,205 (13.83%) | **<0.001** |
| CHF | 32 (4.98%) | 2,290 (4.40%) | 0.476 |
| CKD | 36 (5.60%) | 3,753 (7.20%) | 0.117 |
| **COVID-19 Disease Severity** |  |  |  |
| Hospitalized (General Floor) | 147 (22.86%) | 12,430 (23.86%) | **<0.001** |
| Hospitalized (Critically Ill) | 50 (7.78%) | 3,103 (5.96%) | 0.053 |
| **Vaccination Status** |  |  |  |
| Unvaccinated | 538 (83.67%) |  |  |
| Partially Vaccinated | 5 (0.78%) |  |  |
| Fully Vaccinated | 65 (10.11%) |  |  |
| Fully Vaccinated with Booster | 27 (4.20%) |  |  |
| **Median Household Income Quintile** |  |  |  |
| 1 ($55,275.80) | 346 (53.81%) | 22,745 (43.67%) | **<0.001** |
| 2 ($69,938.40) | 151 (23.48%) | 14,082 (27.03%) | **0.044** |
| 3 ($88,767.60) | 82 (12.75%) | 6,621 (12.71%) | 0.975 |
| 4 ($115,314.60) | 22 (3.42%) | 2, 739 (5.26%) | **0.038** |
| 5 ($250,000) | 27 (4.20%) | 2, 199 (4.22%) | 0.977 |
| **Insurance Status** |  |  |  |
| Private | 212 (32.97%) | 13, 643 (26.19%) | **<0.001** |
| Medicaid | 204 (31.73%) | 21,364 (41.01%) | **<0.001** |
| Medicare | 96 (14.93%) | 10,164 (19.51%) | **0.004** |
| Uninsured | 45 (7.00%) | 2,752 (5.28%) | 0.054 |
| Care Management Organization > Private | 77 (11.98%) | 2,068 (3.97%) | **<0.001** |
| Other | 7 (1.09%) | 622 (1.19%) | 0.807 |
| **Unmet Social Needs** | **N=369** | **N=10,376** |  |
| 0 | 259 (70.19%) | 8,457 (81.51%) | **<0.001** |

**Supplemental Figure 1.** *CORE (N=369) and all COVID+ (N=10,376) patient SDOH screening results. *p<0.05, **p<0.01, ***p<0.001 as compared to CORE.*

*
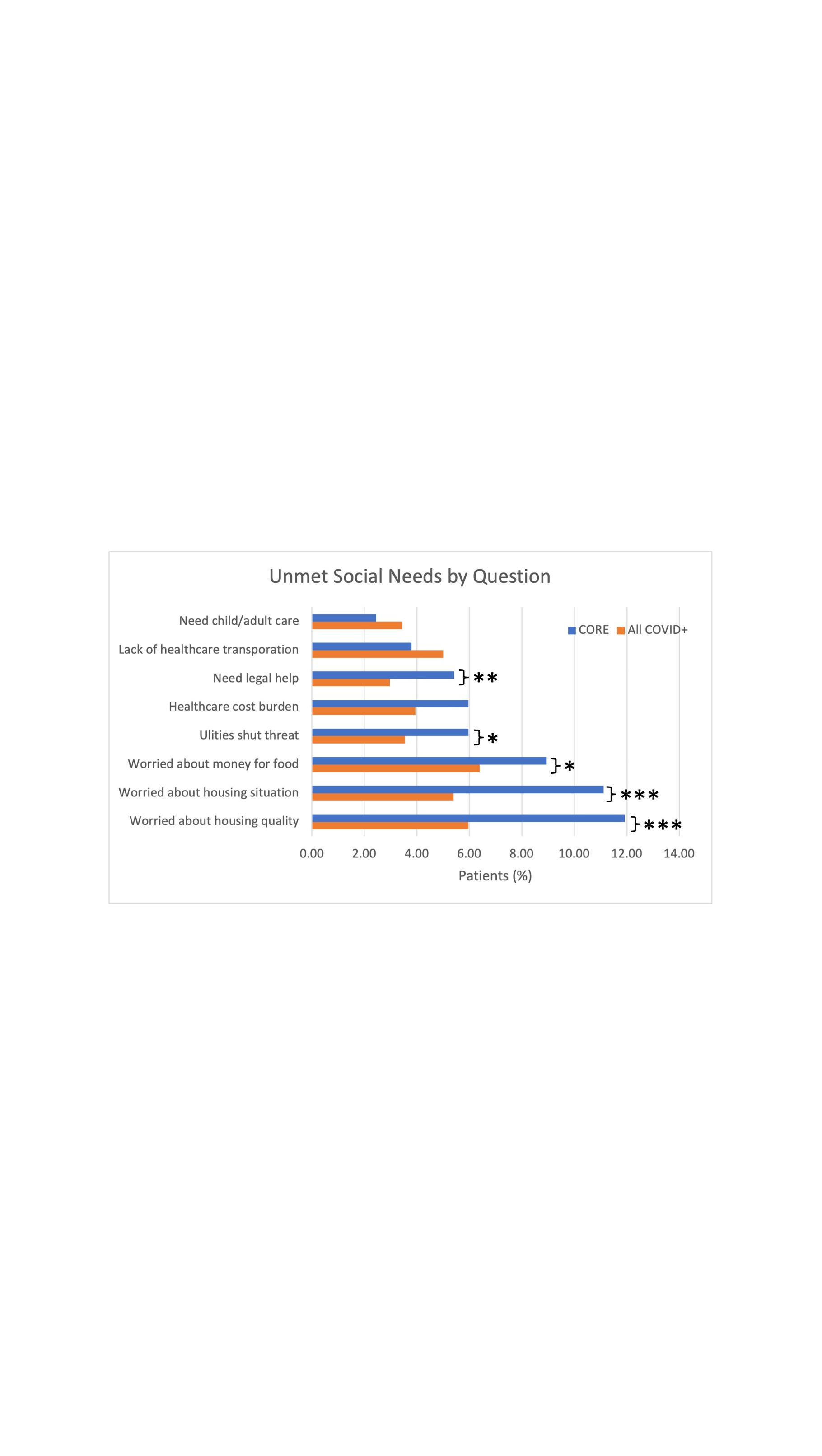
*
